# An analysis of influencing factors of oral frailty in the elderly in the community

**DOI:** 10.1186/s12903-024-03946-y

**Published:** 2024-02-21

**Authors:** Shuying Hu, Xia Li

**Affiliations:** General Practice Ward/International Medical Center Ward, General Practice Medical Center, West China Hospital, Sichuan University, No. 37, Guoxue Lane, Chengdu, 610041 Sichuan Province China

**Keywords:** Oral weakness, Analysis of influencing factors, Elderly, Physical frailty

## Abstract

**Objective:**

This study aimed to investigate the current situation of oral frailty (OF) in the elderly in the community in China and analyse its influencing factors.

**Methods:**

Using convenience sampling, 380 elderly people from three communities in our city were selected as participants in the study. The Oral Frailty Index-8, the Frailty Scale, the Oral Health Assessment Tool, the Mini-nutritional Assessment Scale and the Pittsburgh Sleep Quality Index were used to investigate and analyse OF influencing factors.

**Results:**

In this study, the 380 elderly participants were categorized into three groups: frailty, pre-frailty, and non-physical frailty, based on their responses to the questionnaires.The influencing factor analysis showed that age, gender, education level, frailty score, frailty stage, number of dentures, dry mouth, subjective chewing difficulty, oral health score and sleep quality were the influencing factors of OF in the elderly in the community (R^2^ = 0.712, F = 434.73, *P* < 0.05). The evaluation of the prediction results showed that the frailty score (area under the curve [AUC]: 0.751, 95% confidence interval [CI]: 0.683–0.862), subjective chewing difficulty (AUC: 0.765, 95% CI: 0.655–0.831) and sleep quality (AUC: 0.736, 95% CI: 0.652–0.781) had a higher predictive value for OF.

**Conclusion:**

The main OF influencing factors in the elderly in the community are age, gender, education level, physical frailty (PF) score, PF stage, number of dentures, dry mouth, subjective chewing difficulties, oral health score and sleep quality. Nursing staff should pay attention to the OF of the elderly in the community and take targeted intervention measures in time to reduce and control OF occurrence and progression.

## Introduction

With the aging of the population, the problem of oral diseases in the individuals aged 60 years and above is becoming more prominent [1]. Dental problems, such as tooth loss, dental caries, periodontal disease and dry mouth, can seriously affect the quality of life of the elderly. The concept of oral frailty (OF) has been proposed to describe a type of geriatric syndrome where decreased oral function is accompanied by decreased cognitive and physical function [2]. Oral frailty can cause malnutrition and many other negative health outcomes in the elderly [3, 4]. Studies [5–7] have shown that the incidence of OF in the elderly is 8.1%–53.2%, and the incidence of pre-OF is 33.7%–75.6%. The syndrome is reversible in the early stages of weakness [8], and if intervention measures are actively applied then, further deterioration can be prevented and the occurrence of OF controlled. However, OF often manifests as subtle symptoms, such as increased intake of non-chewing food or difficulty in swallowing, which may not be noticed by carers or elderly people themselves [3].

Physical frailty (PF), as an important aspect of overall frailty, is a high-risk factor for adverse outcomes, such as falls, disability, hospitalisation and death [9]. Studies have shown that the decline of oral function in the elderly may directly lead to PF, which, in turn, affects people’s overall health and quality of life. In the cross-sectional study of Komatsu et al. [10], the correlation between PF indicators (including slow walking speed, reduced grip strength, fatigue, low physical activity and weight loss) and OF of the elderly in the community was evaluated. Thet found that pre-PF (odds ratio (OR) = 2.4, 95% confidence interval (CI) = 1.220–4.750, *P* = 0.012) and pace (the average walking speed of an elderly person in metres per second, measured by a stopwatch) (OR = 0.850, 95% CI = 0.730–0.970, *P* = 0.019) were significantly associated with OF. Hironaka et al. [11] showed that pre-PF (OR = 1.726, 95% CI = 1.202–2.479) was associated with an increased OF risk. The longitudinal study of Iwasaki et al. [12] showed that OF can also cause malnutrition and weight loss in the elderly. In addition, Tanaka et al. [4] found that the risk of PF in elderly people with OF is 2.4 times that of the elderly without OF. It is known that the reduction in teeth, the decrease of tongue pressure, the decrease of the swallowing function and the periodontitis caused by OF are the main causes of overall frailty [13, 14]. Therefore, OF prevention can play an important role in preventing frailty and prolonging the healthy life expectancy of the elderly [15].

Although there have been studies of OF and its influencing factors elsewhere, Chinese research has mainly been focused on the physical, psychological and cognitive frailty of the elderly, and less attention has been paid to OF. Therefore, there is a lack of epidemiological research data on OF in the elderly population in China. At the same time, insufficient attention has been paid to the relationship between oral weakness and physical weakness in China. Therefore, it is of great importance to explore the incidence and influencing factors of OF in the elderly, as well as the correlation between OF and PF, to change the cognition and attitude of medical staff and the elderly themselves to OF and to carry out effective and continuous oral health management in the early stages of OF [16]. The findings may provide a broader idea for the prevention and treatment of frailty in elderly patients. Therefore, the participants in this study are the elderly in the community, and the study investigates the prevalence of OF in the elderly in the community, analyses the influencing factors of OF and discusses the relationship between OF and PF to provide a reference for early assessment, OF prevention and the formulation of targeted nursing measures for nursing staff.

## Materials and methods

### Study subjects

Using the convenience sampling method, the elderly in three communities of our city were selected as study participants. The inclusion criteria were as follows: (1) ≥ 60 years old; (2) long-term residence in the area (for more than 6 months within 1 year); and (3) people with normal language expression and communication skills. The exclusion criteria were as follows: (1) those who had been diagnosed with severe mental disorders, physical disorders and dementia by professional medical institutions; (2) those who could not participate in and complete the questionnaire survey and a physical examination; (3) and those with severe heart, brain, kidney and other organ dysfunction or in the acute phase of the disease. A total of 380 elderly people were finally included in the study, which was approved by the Ethics Committee of the hospital. All subjects gave informed consent and signed a written consent form.

### Study method

A paper questionnaire was distributed on the spot by investigators who were trained in a uniform manner, and the same guidance language was used to help the elderly participants complete it. After completion, the questionnaire was collected in and checked on the spot. If there were missing items or incomplete answers, the questionnaire was returned to the respondent for amendment. The investigator assisted those with poor literacy or vision by reading out the questions and responses. A total of 400 questionnaires were distributed, and 380 valid questionnaires were recovered, with an effective questionnaire recovery rate of 95%.

### Data collection

Demographic data of the participants were collected, including age, gender, smoking history, drinking history, education level, marital status, living status and per capita annual income of the family. Disease-related data was also collected, including number of chronic diseases, payment method of medical expenses, number of teeth, number of dentures, dry mouth issues, subjective chewing difficulties, OF score, PF status, oral health, nutritional status, and sleep quality.

Oral frailty was evaluated using the OF checklist proposed by Tanaka et al. [17]. The checklist consists of 8 items: (1) whether it is harder to eat solid food than it was half a year ago; (2) whether they sometimes choke on tea or soup; (3) whether they have false teeth; (4) whether they have dry mouth symptoms; (5) whether the number social outings has decreased compared with half a year ago; (6) whether they can chew hard food, such as peanuts or pickled radish; (7) whether they have brushed their teeth at least twice a day; and (8) whether they see a dentist at least once a year. The score ranges from 0 to 11 points, with 0–2 points indicating a low OF risk, 3 points indicating a moderate risk and ≥ 4 points indicating a high risk. Receiver operating characteristic (ROC) curve analysis was used to evaluate the predictive value of each of the 8 independent variables for OF occurrence, with an OF score of 4 or higher indicating the existence of OF. The area under the curve (AUC), sensitivity, specificity, positive predictive value and negative predictive value of each variable were calculated with respect to predicting the occurrence of OF.

The study employed several forms of assessment. The Frailty Scale, developed by the International Association for Nutrition, Health and Aging, assesses five key areas: fatigue, resistance, ambulation, illness, and weight loss,each item on the scale is scored 1 point,a total score of 3 or more points indicates the presence of frailty [18]. The Oral Health Assessment Tool (OHAT) was revised by Chalmers et al. [19] and consists of a concise oral health checklist with 8 items: lips, tongue, gingival tissue, saliva, natural teeth, dentures, oral cleaning and toothache. The score for each item is 0 (normal), 1 (lesion) or 2 (abnormal), and the total score is 0–16 points; the higher the total score, the worse the oral health. The Mini-nutritional Assessment Short Form was developed by Rubenstein et al. [20] and contains 6 items: body mass index, recent weight change, acute diseases or major psychological changes, activity ability, neuropsychiatric diseases and food intake. The total score can be 0–14 points, with ≥ 11 points indicating normal nutrition and < 11 points indicating malnutrition. Finally, sleep quality was evaluated using the Pittsburgh Sleep Quality Index [21], which has 7 components: subjective sleep quality, sleep latency, sleep duration, habitual sleep efficiency, sleep disturbances, use of sleeping medication and daytime dysfunction. The total score was 0–21, and the higher the score, the worse the sleep quality: 0–5 for excellent sleep quality, 6–10 indicating good sleep quality, 11–15 representing moderate sleep quality, and 16–21 denoting poor sleep quality.

### Statistical analysis

The sample size was calculated using the G*Power software (version 3.1.9.7, Heinrich Heine University Düsseldorf, Germany). Based on a significance level of 0.05, a power of 0.80 and an effect size of 0.15 for multiple linear regression analysis, the minimum sample size was estimated to be 368. Since 380 elderly people participated in the study, it exceeded the minimum requirement. Statistical analysis was performed using SPSS 26.0 statistical software. Normally distributed measurement data were expressed as (x ± s), and analysis of variance was used for comparison between groups. The count data were expressed as frequency (n) or rate (%). The χ2 test was used for those who met the conditions, and the Fisher exact probability method was used for those who did not meet the conditions. The chi-square test was used for the comparison of categorical variables between groups, and the Fisher exact probability method was used when the expected frequency of any cell was less than five.

Receiver operating characteristic curve analysis was used to evaluate the predictive value of each of the 8 independent variables on the occurrence of OF. The dependent variable was the occurrence of OF, defined as having an OF score of 4 or higher. The independent variables were the 10 factors that were included in the multiple linear regression model.

## Results

### General information

There were 278 community elderly in the frailty group, 137 males and 141 females, with an average age of 77.28 ± 4.67 years and an OF score of 7.28 ± 4.67; there were 58 community elderly in the pre-frailty group, 32 males and 26 females, with an average age of 70.362 ± 4.87 years and an OF score of 6.71 ± 2.03; and there were a total of 44 community elderly people in the non-PF group, 40 males and 4 females, with an average age of 66.50 ± 3.63 years and an OF score of 6.16 ± 1.54. There were statistically significant differences in age, height, sex ratio, residence ratio, living situation, occupation, number of chronic diseases, number of teeth, number of dentures, proportion of dry mouth, subjective chewing difficulties, OF score, oral health score, nutritional status and sleep quality among the three groups (*P* < 0.05). However, there was no significant difference in the other factors (*P* > 0.05) (see Table 1).

**Table 1:** Comparison of general data of the elderly in the community

Item	Physical weakness period (*n* = 278)	Early stage of physical frailty (*n* = 58)	No physical weakness (*n* = 44)	*χ*^*2*^/*Z*/*F* value	*P value*
Age(year, $\overline{\mathrm x}$ ± s)	77.28 ± 4.67	70.362 ± 4.87	66.50 ± 3.63	139.258	< 0.05
Hight(m, $\overline{\mathrm x}$ ± s)	1.63 ± 0.07	1.63 ± 0.07	1.64 ± 0.04	11.052	< 0.05
Weight(kg, $\overline{\mathrm x}$ ± s)	59.42 ± 7.92	64.79 ± 14.52	56.57 ± 9.31	0.725	0.485
Sex(Man/Woman)	137/141	32/26	40/4	26.599	< 0.05
Marriage (Yes/No)	274/4	58/0	43/1	-	0.640
Residence(town/rural aera)	232/46	55/3	29/15	15.004	< 0.05
Smoke(number)	220	50	36	1.583	0.453
Drink(number)	98	23	15	0.467	0.792
Standard of culture(number)				6.362	0.042
Bachelor degree and above	66	9	11		
College for professional training	39	14	20		
Secondary / high school	45	7	0		
Junior high school and below	128	28	13		
Inhabiting information(number)				6.640	< 0.05
Live by oneself	62	21	15		
Cohabiting with others	216	37	29		
Family annual income(RMB)				2.835	0.242
< 10,000	16	4	3		
10000 ~ 30000	80	19	18		
> 30000	182	35	23		
Occupation				0.534	< 0.05
Farmer	102	23	16		
Retiree	103	21	18		
Miscellaneous	73	14	10		
Number of chronic diseases(number)				40.082	< 0.05
0	23	13	14		
1	67	21	18		
≥ 2	188	24	12		
Number of teeth[number,M(P25,P75)]	14(2.3, 20.1)	22(18.0, 23.1)	25(22.0, 26.0)	8.216	< 0.05
Number of dentures[number,M(P25,P75)]	7(3.0, 21.0)	3(1.0, 18.0)	0(0, 2.0)	6.772	< 0.05
Xerostomia(number)	132	15	5	26.349	< 0.05
Subjective chewing difficulty(number)	109	13	3	21.461	< 0.05
OF score($\overline{\mathrm x}$ ± s)	7.28 ± 4.67	6.71 ± 2.03	6.16 ± 1.54	6.913	< 0.05
Oral Health Score[M(P25,P75)]	6.0(3.0, 7.0)	4.0(2.0, 6.0)	2.0(1.0, 4.0)	7.748	< 0.05
Nutritional Status(number)				7.358	< 0.05
Normal	138	34	31		
Dystrophy	140	24	13		
Sleep Quality(number)				24.150	< 0.05
Worse	112	10	6		
General	70	17	7		
Good	96	31	31		

*OF* Oral frailty

### Analysis of the influencing factors of oral frailty scores of the elderly in the community

To further explore the influencing factors of OF, multiple linear regression analysis was used to find out meaningful suspicious risk factors. Stepwise regression analysis was performed with the OF score of the respondents as the dependent variable and the collected variables as the independent variables. The stepwise regression method was used to include and exclude the independent variables (α_entry_ = 0.05, α_removal_ = 0.1), and the influencing factors with interaction were eliminated. The results showed that a total of 10 variables could be included, namely age, gender, education level, frailty stage, frailty score, number of dentures, dry mouth, subjective chewing difficulties, oral health score and sleep quality, and the other variables were to be excluded. The model R^2^ = 0.767 indicates that these 10 variables can explain 76.7% of the factors affecting the OF score, and F = 434.73, *P* < 0.05, indicates that the dependent variable and the 10 variables fit well; the Debin–Watson index is 1.985, which shows that there is no correlation between the independent variables of the model. The significance test results of the 10 independent variables in the model were all *P* < 0.05, which proved that the 10 independent variables were statistically significant in the model and should be retained. In addition, the variance inflation factor values of the 10 independent variables are all less than 10, so there is no collinear relationship between the respective variables. The multiple linear regression equation obtained by fitting is: Y = 14.614 + 0.336X_1_ + 0.431X_2_ + 0.276X_3_ + 0.518X_4_ + 0.183X_5_ + 0.389X_6_ + 0.517X_7_ + 0.640X_8_ + 0.245X_9_ + 0.483X_10_, where Y is the OF score, X_1_ is age, X_2_ is sex, X_3_ is standard of culture, X_4_ is PF score, X_5_ is physical weakness period, X_6_ is number of dentures, X_7_ is xerostomia, X_8_ is subjective chewing difficulty, X_9_ is oral health score and X_10_ is sleep quality (see Table 2).

**Table 2:** Regression model of influencing factors of OF score

Variable	Standard error	Partial regression coefficient	Standardized Partial Regression Coefficient	*P* value	*95%CI*	VIF
Constant	14.614	-	-	< 0.05	-	-
Age	1.380	0.336	0.236	< 0.05	0.235 ~ 0.422	2.158
Sex	1.231	0.431	0.301	< 0.05	0.235 ~ 0.422	1.491
Standard of culture	1.068	0.276	0.193	< 0.05	0.165 ~ 0.361	1.408
Physical frailty score	0.090	0.518	0.362	< 0.05	0.457 ~ 0.713	2.432
Physical weakness period	0.183	0.183	0.128	< 0.05	0.112 ~ 0.223	2.594
Number of dentures	1.727	0.389	0.272	< 0.05	0.236 ~ 0.531	1.948
Xerostomia	1.333	0.517	0.361	< 0.05	0.421 ~ 0.712	1.727
Subjective chewing difficulty	1.318	0.640	0.447	< 0.05	0.532 ~ 0.716	1.208
Oral Health Score	1.975	0.245	0.171	< 0.05	0.215 ~ 0.334	1.454
Sleep Quality	1.354	0.483	0.337	< 0.05	0.362 ~ 0.533	1.586

*R* = 0.876, R2 = 0.767, adjust R2 = 0.712, F = 434.73, *P* < 0.05 *OF* oral frailty, *VIF* variance inflation factor Age: the age of the elderly in years Sex: 1 for man, 2 for woman Standard of culture: the highest level of education attained by the elderly, 1 for bachelor degree and above, 2 for college for professional training, 3 for secondary / high school, 4 for junior high school and below Physical frailty score: the score of the FRAIL scale, ranging from 0 to 5 Physical weakness period: the stage of physical frailty according to the FRAIL scale, 1 for physical weakness period, 2 for early stage of physical frailty, 3 for no physical frailty Number of dentures: the number of dentures worn by the elderly Xerostomia: whether the elderly have dry mouth symptoms, 1 for yes, 2 for no Subjective chewing difficulty: whether the elderly feel harder to eat hard food than half a year ago, 1 for yes, 2 for no Oral Health Score: the score of the OHAT, ranging from 0 to 16 Sleep Quality: the score of the PSQI, 1 for worse, 2 for general, 3 for good Data source: This table is based on the data collected from 380 community-dwelling elderly people in Yangjiang City, Guangdong Province, China, from September to November 2023

### Evaluation of the predictive value of each influencing factor on the occurrence of oral frailty

**Fig. 1:**
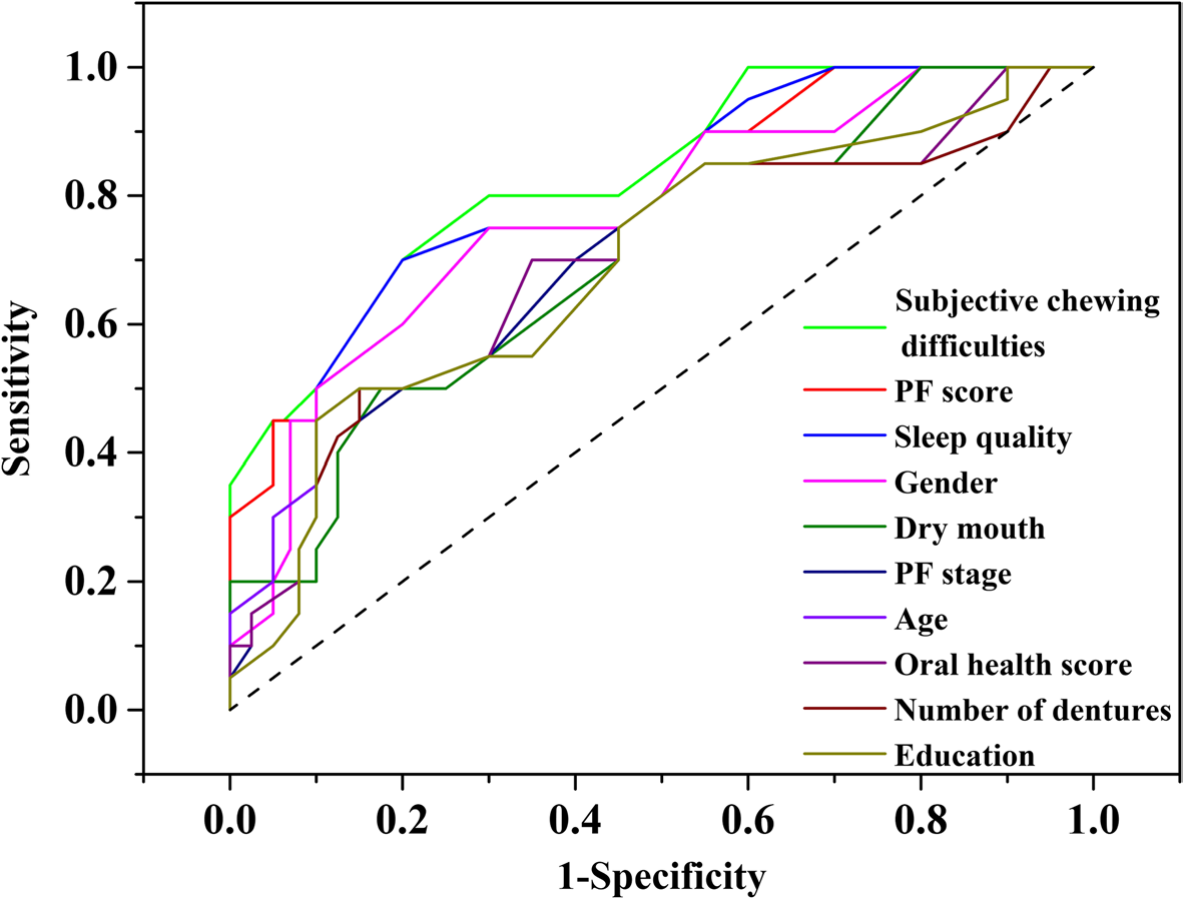
ROC curve diagram

The results showed that the 10 variables had certain predictive values for the occurrence of OF. Among them, the frailty score, subjective chewing difficulty and sleep quality had higher predictive values for OF. The AUC of frailty predicting OF was 0.751 (95% CI: 0.683–0.862); the AUC of subjective chewing difficulty in predicting OF was 0.765 (95% CI: 0.655–0.831); and, the AUC of sleep quality in predicting OF was 0.736 (95% CI: 0.652–0.781). The predictive value of other factors for the occurrence of OF is shown in Table 3, and the ROC curve is shown in Fig. 1.

**Table 3:** Evaluation of the predictive value of each influencing factor on the occurrence of OF

Item	Accuracy	Sensitivity	Specificity	AUC	*95%CI*
Age	0.612	0.632	0.633	0.611	0.532 ~ 0.651
Sex	0.611	0.643	0.731	0.696	0.581 ~ 0.726
Standard of culture	0.631	0.731	0.687	0.595	0.553 ~ 0.642
Physical frailty score	0.812	0.843	0.844	0.751	0.683 ~ 0.862
Physical weakness period	0.641	0.674	0.712	0.592	0.527 ~ 0.634
Number of dentures	0.731	0.734	0.755	0.591	0.513 ~ 0.612
Xerostomia	0.812	0.835	0.851	0.589	0.553 ~ 0.731
Subjective chewing difficulty	0.873	0.874	0.831	0.765	0.655 ~ 0.831
Oral Health Score	0.723	0.745	0.752	0.591	0.541 ~ 0.632
Sleep Quality	0.789	0.791	0.812	0.736	0.652 ~ 0.781

*OF* Oral frailty, *PPV* Positive predictive value, *NPV* Negative predictive value OF occurrence: having an OF score of 4 or higher. Age: the age of the elderly in years Sex: 1 for man, 2 for woman Standard of culture: the highest level of education attained by the elderly, 1 for bachelor degree and above, 2 for college for professional training, 3 for secondary / high school, 4 for junior high school and below Physical frailty score: the score of the FRAIL scale, ranging from 0 to 5 Physical weakness period: the stage of physical frailty according to the FRAIL scale, 1 for physical weakness period, 2 for early stage of physical frailty, 3 for no physical frailty Number of dentures: the number of dentures worn by the elderly Xerostomia: whether the elderly have dry mouth symptoms, 1 for yes, 2 for no Subjective chewing difficulty: whether the elderly feel harder to eat hard food than half a year ago, 1 for yes, 2 for no Oral Health Score: the score of the OHAT, ranging from 0 to 16 Sleep Quality: the score of the PSQI, 1 for worse, 2 for general, 3 for good Data source: This table is based on the data collected from 380 community-dwelling elderly people in Yangjiang City, Guangdong Province, China, from September to November 2023

## Discussion

This study found that age, gender, education level, frailty score, frailty stage, number of dentures, dry mouth, subjective chewing difficulty, oral health score and sleep quality were the influencing factors of OF. According to the standardised partial regression coefficients in the model, the order of influence of the 10 independent variables on the OF score was as follows: subjective chewing difficulty > physical weakness score > dry mouth > sleep quality > gender > number of dentures > age > education level > oral health score > physical weakness stage. This study found that the prevalence of OF among the elderly in the community increased with age, which is consistent with other studies [22]. The possible reason is that, with the increase in age, the alkaline phosphatase activity of periodontal ligament cells and their regeneration ability and osteogenic activity will decrease, and the physiological atrophy of gingiva and the demineralisation and softening of cementum will occur in the elderly, resulting in periodontitis, dental caries and other diseases, leading to OF [23]. In addition, the risk of OF in elderly women is higher than in men. The possible reason is that the development of permanent teeth in girls takes place earlier than it does in boys, so the time of chewing wear and bacterial corrosion is longer [24]. At the same time, the gum is the target organ of estrogen and postmenopausal elderly women have low estrogen levels and more bone calcium loss. Problems such as alveolar osteoporosis and atrophy, reduced saliva secretion in oral mucosa, a slowed flow rate and increased vascular permeability, causing xerostomia, dental caries and periodontal disease, can occur [25], leading to oral weakness.

The link between physical and oral frailty may be attributed to the fact that physical symptoms, such as decreased physiological reserve function, decreased activity and decreased physical strength are related to a reduction in the social range of the elderly, a reduction in oral communication opportunities and a reduction in oral and maxillofacial muscle and tongue movement, which are correlated with decreased tongue pressure, weakness in chewing, difficulty in swallowing, and slowed tongue movement, all of which can result in OF [9]. The number of dentures is another influencing factor of OF in the elderly. The possible reason is that the bone tissue of the elderly wearing dentures absorbs rapidly, the base tissue surface lacks close adhesion to the mucosa of the bearing area and the mucosa is easily colonised by oral fungi. Problems such as reduced saliva volume, improper denture cleaning and food impaction can also cause denture-related diseases [26], increasing the risk of OF. Therefore, nursing staff should provide personalised denture care guidance for elderly people with dentures, show them how to use them correctly, encourage them to establish good denture care habits, urge them to regularly review their dentures and oral health and use multimedia and internet tools to track and follow up their oral hygiene [27]. The elderly with low oral health scores are more likely to have problems, such as reduced saliva volume, reduced tongue pressure, chewing disorders and dysphagia, which will accelerate the deterioration of OF [28]. Suzuki et al. [29] found that daily oral health management services provided by oral health nurses can improve the swallowing function of the elderly on the ward and promote oral health. Therefore, providing convenient daily oral health management services for the elderly may help to prevent OF.

Studies have found that elderly people with chronic diseases who take a variety of drugs for a long time often develop oral dryness [4], which is associated with dental caries and chewing and swallowing function decline, affecting the oral function of the elderly [30]. Numerous studies have also shown that elderly patients with chronic diseases, such as cognitive impairment [31], Alzheimer's disease [32], cardiovascular disease [10], stroke [11] and diabetes [33] are more likely to suffer from OF. The current study also found that poor sleep quality is one of the risk factors for OF. Since sleep quality is related to PF, the worse the sleep quality, the higher the degree of PF in the elderly [34], which, in turn, affects their oral health. Thus, PF is associated with OF in the elderly in the community.

This study has several shortcomings. First, it has a cross-sectional study design, and the influencing factors discussed cannot be used as the basis for causal inference. In addition, this is a single-centre study, and it is difficult to ensure that the baseline is consistent when the cohort is compared in groups, and patients are likely to have other complications that may affect their prognosis. Moreover, at present, the evaluation of OF in clinical practice is mostly performed using scales that rely on signs and the subjective feelings of patients, and there is a lack of objective data measured by modern instruments and standardised curative effect criteria. Therefore, continuing the research and forming objective and uniform observation indicators are necessary. This study used the OHAT to assess the oral health status of the elderly, which does not measure the level of caries, periodontal affectation, gingival insertion or dental mobility of the remaining teeth, and these particular factors may also affect the oral function and quality of life of the elderly. Therefore, future studies should use more comprehensive oral health assessment tools that include the state of the remaining teeth, such as the decayed, missing and filled teeth index, the community periodontal index, and the oral hygiene index-simplified.

## Conclusion

In summary, the main OF influencing factors in the elderly in the community include age, gender, education level, PF score, PF stage, number of dentures, dry mouth, subjective chewing difficulties, oral health score and sleep quality.

The findings suggest that carers should regularly check the oral status of the elderly in the community, find out the potential OF population by using the Oral Frailty Index-8 and provide individualised and comprehensive oral health guidance for the elderly. Understanding the factors associated with OF and their predictive value could help to prevent or delay the occurrence and development of OF and improve the oral health level of the elderly in the community, and, consequently, their overall health.

## Data Availability

All data generated or analysed during this study are included in this article. Further enquiries can be directed to the corresponding author.
